# Central Serous Chorioretinopathy in Parallel With Onset and Relapses of Minimal Change Nephrotic Syndrome: A 28-Year Case Follow-Up

**DOI:** 10.7759/cureus.102426

**Published:** 2026-01-27

**Authors:** Toshihiko Matsuo, Takehiro Tanaka, Jun Wada

**Affiliations:** 1 Department of Ophthalmology, Graduate School of Interdisciplinary Science and Engineering in Health Systems, Okayama University, Okayama, JPN; 2 Department of Pathology, Graduate School of Medicine, Dentistry, and Pharmaceutical Sciences, Okayama University, Okayama, JPN; 3 Department of Nephrology, Rheumatology, Endocrinology, and Metabolism, Graduate School of Medicine, Dentistry, and Pharmaceutical Sciences, Okayama University, Okayama, JPN

**Keywords:** central serous chorioretinopathy, corticosteroid, cyclosporine, fluorescein angiography, minimal change disease, minimal change nephrotic syndrome, photoreceptor ellipsoid zone, renal biopsy, steroid-induced retinal pigment epitheliopathy, steroid pulse therapy

## Abstract

Central serous chorioretinopathy is an idiopathic disease that manifests as one or several localized, small, dome-shaped serous retinal detachments on fundus examination. The pathophysiology involves fluid leakage from the choroidal capillaries, known as the choriocapillaris, into the subretinal space through sites of damage in the retinal pigment epithelium. This case report discusses the underlying causes of central serous chorioretinopathy-like findings in minimal change nephrotic syndrome.

The patient was a 33-year-old woman who developed nephrotic syndrome that was confirmed to be minimal change disease by renal biopsy. She experienced two major relapses of nephrotic syndrome at the ages of 36 and 41 years. She also had a minor relapse at the age of 37 years, five months after the first major relapse at the age of 36 years, as well as four additional minor relapses at the ages of 44, 46, 50, and 51 years. The onset of central serous chorioretinopathy-like manifestations, which were localized to the left eye, occurred three months after the initial onset of nephrotic syndrome at the age of 33 years. Two subsequent episodes of relapse of central serous chorioretinopathy-like manifestations were observed in both eyes at intervals of five months and one month, respectively, after major relapses of nephrotic syndrome at the ages of 36 and 41 years. Thereafter, she did not develop further central serous chorioretinopathy-like manifestations.

She discontinued oral prednisolone at the age of 54 years and experienced no further relapses of nephrotic syndrome through her latest visit at the age of 61 years. She maintained normal renal function and good visual acuity in both eyes. The long-term, consistent temporal association between episodes of central serous chorioretinopathy and the onset and relapses of minimal change nephrotic syndrome is strongly supported by longitudinal clinical observations spanning 28 years. This parallel course suggests a possible shared pathophysiological mechanism or common triggering factors underlying both diseases.

## Introduction

Central serous chorioretinopathy is an independent disease entity that is generally not associated with systemic manifestations. A hallmark of the disease is fluid leakage into the subretinal space from the choriocapillaris through one or several damaged sites of the retinal pigment epithelium, as evidenced by fundus examinations, including fluorescein angiography [1]. The subretinal fluid accumulation manifests as one or several areas of small, dome-shaped serous retinal detachment in the posterior pole of the fundus, including the macular area. A similar manifestation has been described, for instance, in systemic lupus erythematosus itself [2], nephrotic syndrome associated with systemic lupus erythematosus [2,3,4], and hypertension associated with membranous nephropathy [5]. Accordingly, the known or hypothesized links between central serous chorioretinopathy and systemic factors include corticosteroid exposure, stress, vascular endothelial dysfunction, and altered vascular permeability [1].

From an anatomical viewpoint, the capillaries of the choroid of the eye, known as the choriocapillaris, have endothelial cell fenestrations that facilitate fluid filtration into the space lined by the Bruch membrane, which consists of the layered basement membranes of the choriocapillaris and retinal pigment epithelial cells [6,7]. Choroidal arterioles enter almost perpendicularly into anastomosing, multi-lobular networks of large-caliber capillaries in the choriocapillaris to nourish retinal pigment epithelial cells, which support photoreceptor outer segments. The glomerulus of the kidney is a globular structure composed of a vascular capillary loop lined by capillary endothelial cells, which also possess fenestrations for enhanced filtration into the glomerular capsule [8]. The afferent arteriole abruptly becomes a long, convoluted capillary loop, which then connects to the efferent arteriole as it exits the glomerulus. The glomerular capillary structure supports efficient, high-pressure filtration of fluid as well as small molecules from the blood, which contains red blood cells and large molecules such as proteins.

The structural similarity between renal glomerular capillaries and the eye’s choriocapillaris [9] suggests a pathophysiological link in which glomerular diseases may be associated with choriocapillaris disorders occurring concurrently. As an example of simultaneous renal and choroidal inflammation, choriocapillaris inflammation and obstruction may occur in association with rapidly progressive glomerulonephritis, which represents glomerular capillary obliteration caused by inflammation [10]. In antineutrophil cytoplasmic antibody (ANCA)-associated vasculitis, glomerulonephritis can occur in association with acute posterior multifocal placoid pigment epitheliopathy (APMPPE), which has a pathological basis of obstruction of choriocapillaris leaflets by inflammation [11,12]. Furthermore, immunoglobulin A (IgA) nephropathy has been reported to occur in association with Vogt-Koyanagi-Harada disease, which is characterized by choroidal inflammation caused by an autoimmune attack on melanocytes [13].

In nephrotic syndrome, plasma proteins are lost into the urine, and patients present with peripheral edema around the eyes and in the lower limbs as a result of hypoalbuminemia. Nephrotic syndrome is classified into two categories, primary and secondary, based on the absence or presence of underlying diseases such as systemic lupus erythematosus [14]. In primary nephrotic syndrome, renal biopsy reveals no pathological changes or immune deposits in the glomeruli by light microscopic observation, a condition referred to as minimal change disease [14]. To date, there has been an accumulation of case reports describing nephrotic syndrome in association with central serous chorioretinopathy-like manifestations [15,16]. Since both central serous chorioretinopathy and nephrotic syndrome are characterized by relapsing and remitting courses, long-term longitudinal clinical observations may provide insight into their underlying relationship. In this report, we describe a long-term follow-up of a patient with minimal change nephrotic syndrome who repeatedly exhibited central serous chorioretinopathy-like manifestations during changes in the dosage of oral prednisolone.

## Case presentation

**Figure 1:**
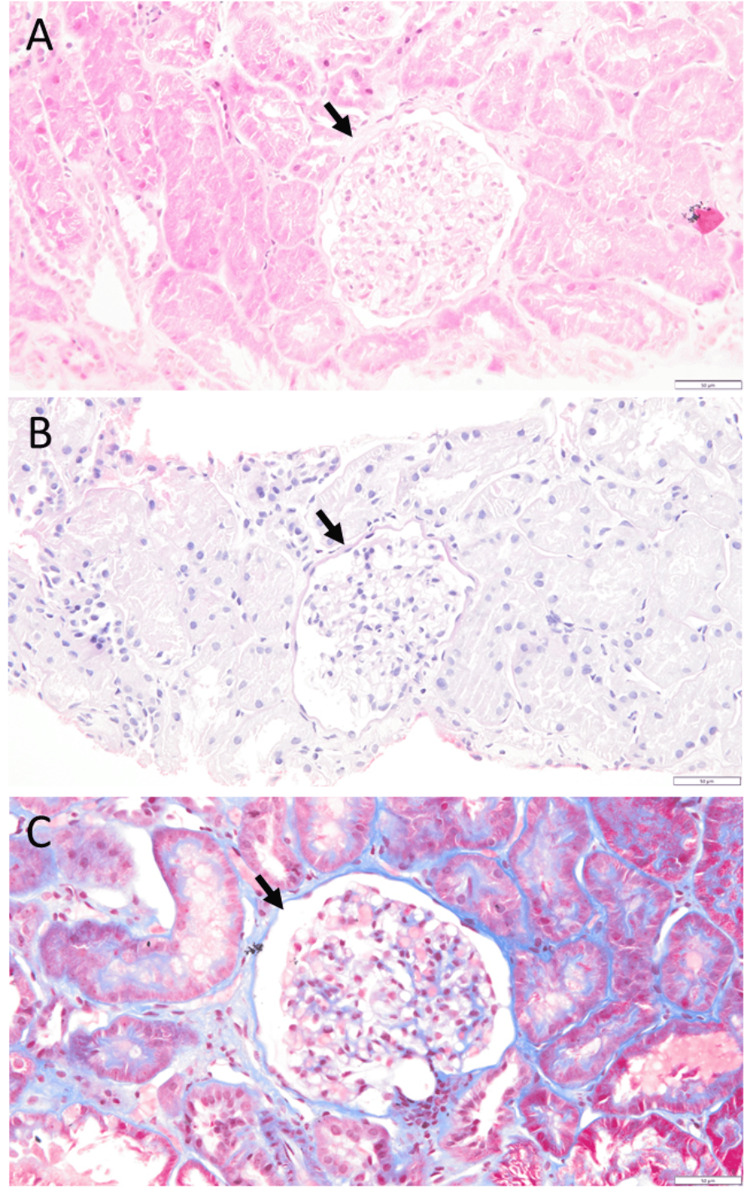
Pathology images of renal biopsy Renal biopsy at the onset of nephrotic syndrome at age 33 years. No pathological changes in the glomerulus (arrows) or renal tubules are observed in hematoxylin-eosin (A), periodic acid-Schiff (B), or Masson trichrome (C) stains. Scale bar = 50 µm

**Figure 2:**
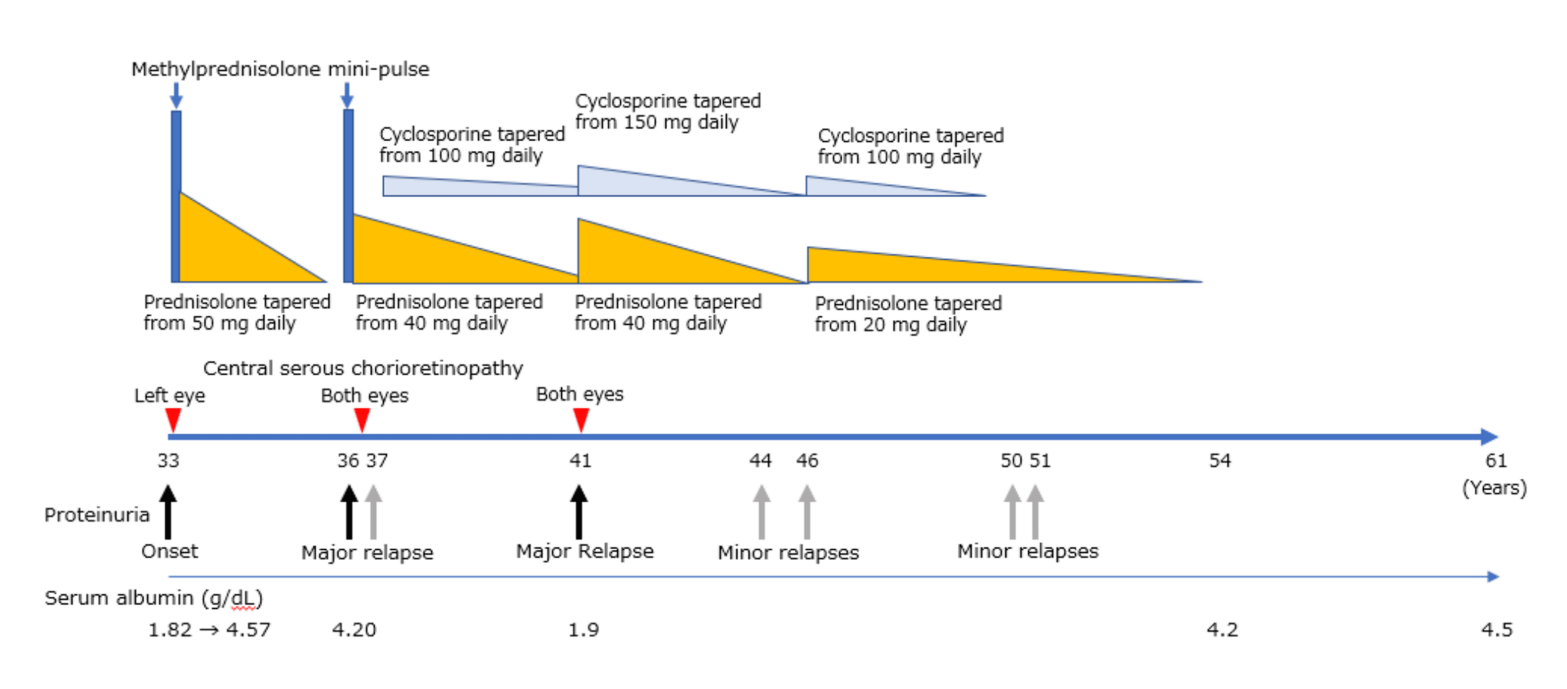
Clinical course chart Clinical course of the patient, showing the onset and relapses of nephrotic syndrome and the treatment, together with the onset and relapses of central serous chorioretinopathy. The initial and final daily doses of oral prednisolone and oral cyclosporine are accurately depicted, while their tapering processes are roughly described. Methylprednisolone mini-pulse indicates a three-day course of intravenous methylprednisolone 500 mg daily

A 33-year-old woman noticed facial and bilateral hand edema, together with a two-week history of epigastric discomfort and an abnormal urine odor. At a physician visit, a urine test-tape examination showed 3+ protein, and blood examinations revealed a decrease in total protein to 4.5 g/dL (normal range: 6.6-8.1), a decrease in albumin to 1.8 g/dL (normal range: 4.1-5.1), and an elevation in total cholesterol to 343 mg/dL (normal range: 142-248). She was referred to a university hospital and was diagnosed with minimal change nephrotic syndrome (Figures 1A, 1B, 1C), based on a renal biopsy. Five glomeruli examined showed no cellular proliferation and no crescent formation, hyalinization, or sclerosis. Immunostaining of biopsy specimens showed no deposition of immunoglobulin G (IgG), IgA, IgM, C3, C1q, or fibrin. She underwent one course of corticosteroid mini-pulse therapy with intravenous methylprednisolone 500 mg daily for three days. Oral prednisolone was then administered at 50 mg daily and tapered (Figure 2).

As for her history, she had experienced Streptococcal infection, which had repeatedly resulted in purpura with nephritis and arthritis at the age of four to six years. She had undergone a bilateral tonsillectomy at the age of six years. She had delivered two healthy babies with no complications at the ages of 29 and 31 years. Her father had died of chronic renal failure at the age of 61 years.

**Figure 3:**
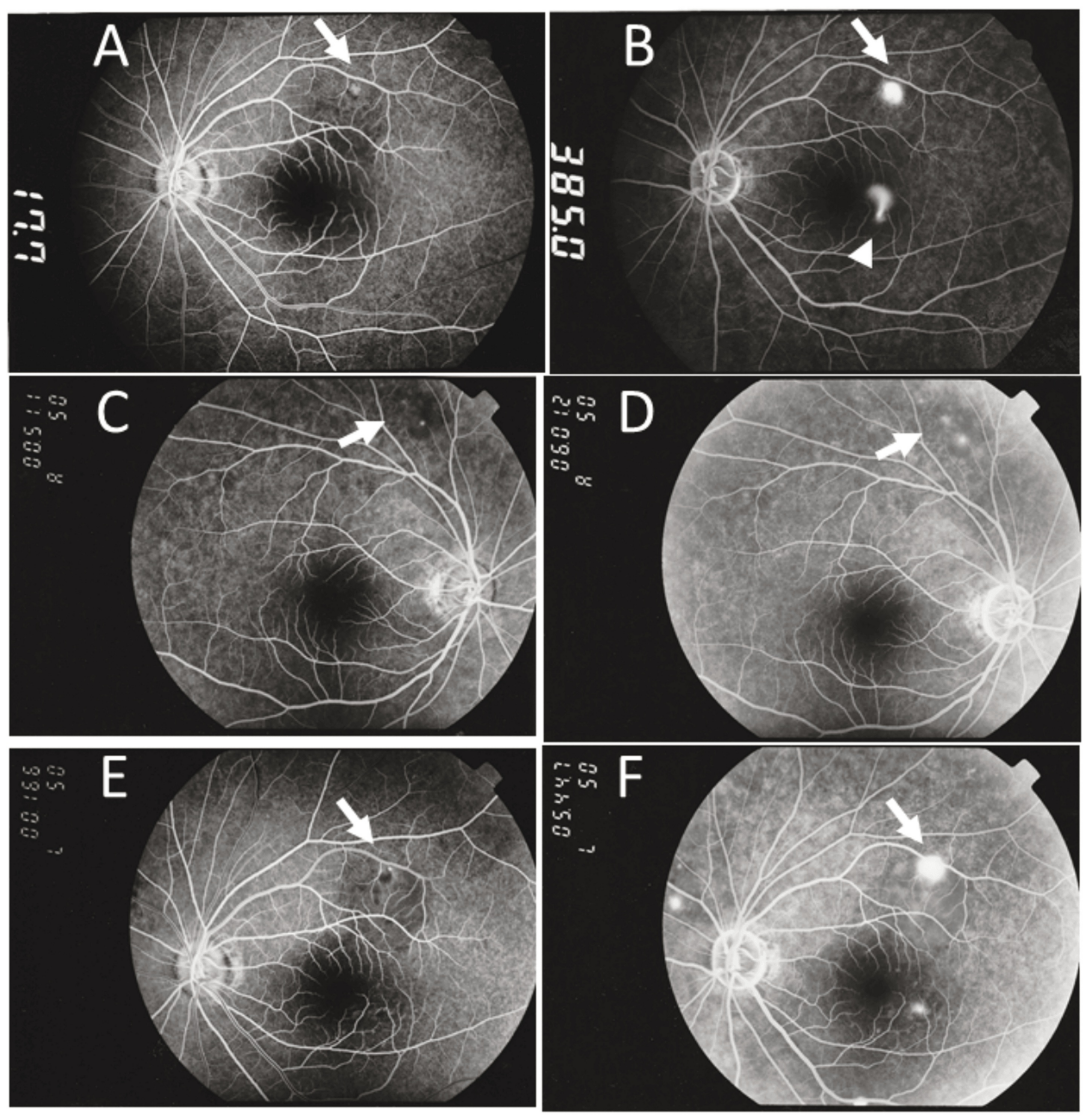
Fluorescein angiography at age 33 and 36 years Early-phase (A) and late-phase (B) fluorescein angiograms in left eye obtained at the initial ophthalmology visit, three months after the onset of nephrotic syndrome at 33 years of age, show spot-like leakage (arrows, A, B) and fountain-like leakage (arrowhead, B). Early-phase (C, right eye; E, left eye) and late-phase (D, right eye; F, left eye) fluorescein angiograms obtained five months after relapse of nephrotic syndrome at 36 years of age show spot-like leakage in the late phases (arrows). The numeric values in each panel indicate the elapsed time in seconds from fluorescein dye injection into the antecubital vein

Three months after corticosteroid administration, she noticed photophobia in the left eye and was referred to an ophthalmologist. At that time, she was taking oral prednisolone 50 mg every other day. The best-corrected visual acuity measured in decimal units was 2.0 in the right eye and 1.5 in the left eye. The intraocular pressure was 13 mmHg in both eyes. Slit-lamp examination revealed no aqueous or vitreous cells. Funduscopic examination showed a normal right eye, whereas the left eye demonstrated a flat serous retinal detachment at the posterior pole. Fluorescein angiography disclosed a fountain-like leakage point near the fovea, along with another slow leakage near the upper vascular arcade (Figures 3A, 3B). Observation was chosen over laser photocoagulation because the visual acuity in the left eye was good. After three months, when oral prednisolone had been reduced to 35 mg every other day, the serous retinal detachment in the left eye subsided.

Oral prednisolone was slowly tapered and discontinued two years and two months later, when she was 35 years old. Thereafter, she monitored urine protein daily at home using urine test-tapes in the early morning and remained free of proteinuria for eight months. At the age of 36 years, she detected 2+ urine protein on one day, followed by 4+ urine protein the next day. At that time, she did not have any symptoms, including peripheral edema or common cold-like symptoms.

At the age of 36 years, during this major relapse of nephrotic syndrome, her height was 158 cm, weight was 56 kg, and blood pressure was 108 mmHg systolic and 68 mmHg diastolic. Physical examination revealed no abnormalities. Blood examinations showed normal total protein and albumin levels (Table 1). She underwent a second course of corticosteroid mini-pulse therapy with methylprednisolone 500 mg daily for three days, followed by oral prednisolone tapered from 40 mg daily. Five months after resumption of corticosteroid therapy, she noticed micropsia in the left eye and developed a relapse of serous retinal detachment at the posterior pole of the left eye when oral prednisolone was 22.5 mg daily. The best-corrected visual acuity was 2.0 in the right eye and 1.2 in the left eye. The intraocular pressure was 13 mmHg in both eyes. Fluorescein angiography disclosed a tiny leakage superior to the upper vascular arcade in the right eye (Figures 3C, 3D), and two leakage points at the same sites as in the previous episode in the left eye (Figures 3E, 3F). After three months, the serous retinal detachment subsided when oral prednisolone was reduced to 17.5 mg daily.

**Table 1:** Blood examinations during the disease course ^*^Normal ranges for LD values were 236-455 IU/L, determined by a different method in earlier years Values at 61 years correspond to in-house normal ranges LD: lactate dehydrogenase; AST: aspartate aminotransferase; ALT: alanine aminotransferase; eGFR: estimated glomerular filtration rate; n.d.: not determined

Parameter	Unit	Normal range	33 years (Onset)	33 years (5 months from onset)	36 years (first relapse)	37 years (second relapse)	40 years (6 months before the third relapse)	41 years (third relapse)	54 years (prednisolone discontinued)	61 years (latest visit)
Red blood cells	x 10^6^/µL	3.86-4.92	5.09	4.22	4.77	4.28	4.37	5.08	4.13	4.21
Platelets	x 10^3^/µL	158-348	276	220	269	244	252	280	221	236
White blood cells	x 10^3^/µL	3.30-8.60	8.9	9.3	8.4	11.5	5.54	13.6	5.03	5.19
Hemoglobin	g/dL	11.6-14.8	15.6	13.4	14.8	13.9	13.6	16.0	12.8	13.3
Hematocrit	%	35.1-44.4	46.8	39.6	44.9	40.3	42.9	47.5	38.4	39.5
Total protein	g/dL	6.6-8.1	4.48	6.58	6.89	5.84	6.9	4.1	6.3	6.9
Albumin	g/dL	4.1-5.1	1.82	4.57	4.20	3.77	4.4	1.9	4.2	4.5
LD	U/L	124-222	362^*^	348^*^	294^*^	307^*^	191	190	155	161
AST	U/L	13-30	16	11	14	13	32	19	15	17
ALT	U/L	7-23	9	8	10	10	32	13	9	10
Total bilirubin	mg/dL	0.40-1.50	0.49	0.69	0.84	0.78	0.60	0.53	0.80	0.72
Urea nitrogen	mg/dL	8.0-20.0	12.7	10.4	12.1	6.4	9.9	15.3	10.1	10.4
Creatinine	mg/dL	0.46-0.79	0.55	0.60	0.60	0.68	0.69	0.78	0.61	0.67
eGFR	mL/min/1.73 m^2^	60 or greater	n.d.	n.d.	n.d.	n.d.	66.4	n.d.	78.4	68.3
Total cholesterol	mg/dL	142-248	359	209	n.d.	271	210	438	184	211
Postprandial glucose	mg/dL	<140	n.d.	n.d.	n.d.	n.d.	111	n.d.	104	97

One year and one month later, at 37 years of age, she experienced a minor relapse of proteinuria while taking oral prednisolone 20 mg daily. Oral cyclosporine 100 mg daily was added to her current prednisolone dose, leading to the resolution of proteinuria. Three and a half years later, at age 41, she experienced a second major relapse while taking oral prednisolone 5 mg daily and cyclosporine 40 mg daily. Blood tests showed low total protein and albumin levels (Table 1). Oral prednisolone was increased to 40 mg daily, and cyclosporine was increased to 150 mg daily. One month later, she noticed blurred vision in both eyes while taking oral prednisolone 30 mg daily. The best-corrected visual acuity was 1.5 in both eyes, and intraocular pressure was 15 mmHg in both eyes.

**Figure 4:**
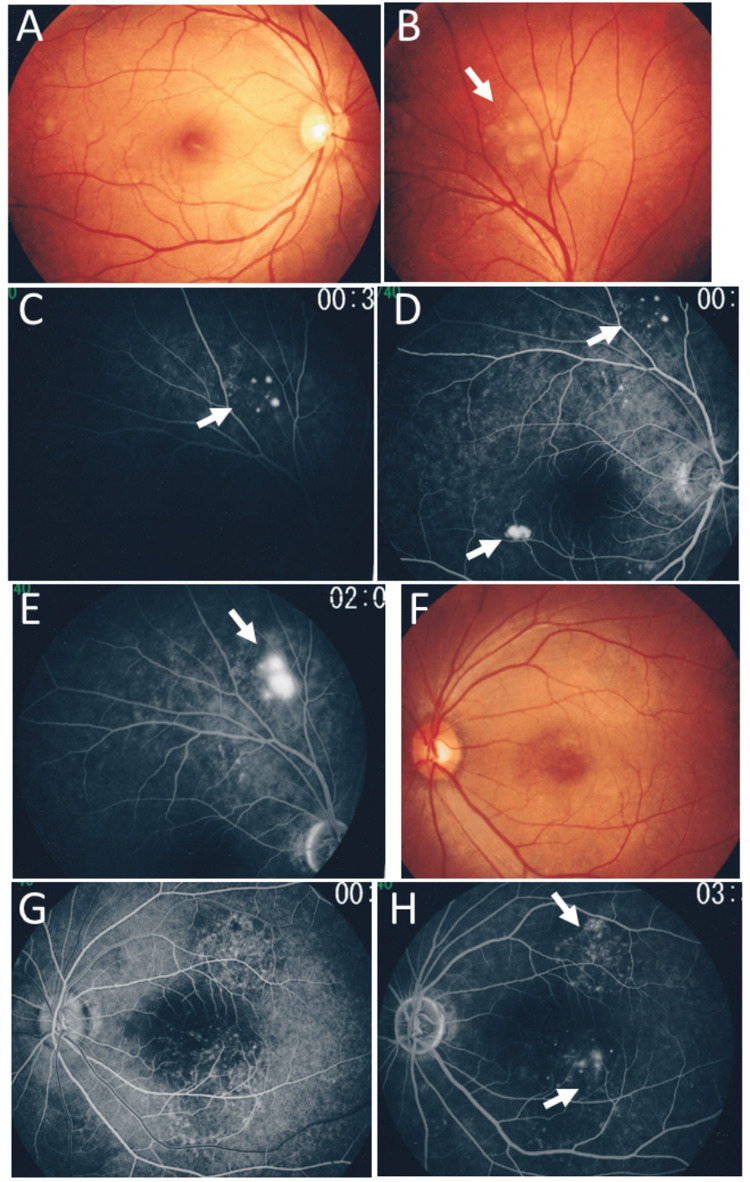
Fundus photographs and fluorescein angiography at age 41 years Fundus photographs (A, B: right eye, F: left eye) and early phases (C, D: right eye, G: left eye) and late phases (E: right eye, H: left eye) of fluorescein angiograms in a month of the relapse of nephrotic syndrome at age 41 years. Note round serous retinal detachment (arrow, B) in the right eye and enlarging spot-like leakages in the right eye (arrows, C, D, E) and in the left eye (arrows, H). Numeric values in C, D, E, G, and H indicate a timer (minutes : seconds) to show the passage of time from fluorescein dye injection at the antecubital vein

Fundus examinations revealed that the right eye had a localized, flat-shaped serous retinal detachment superior to the upper vascular arcade (Figures 4A-4E), at the same site as a previous episode, while the left eye showed two areas of localized, flat-shaped serous retinal detachment superior and inferior to the macula (Figures 4F-4H). At a six-month follow-up visit, there was no serous retinal detachment, with oral prednisolone reduced to 5 mg daily and cyclosporine to 40 mg daily.

**Figure 5:**
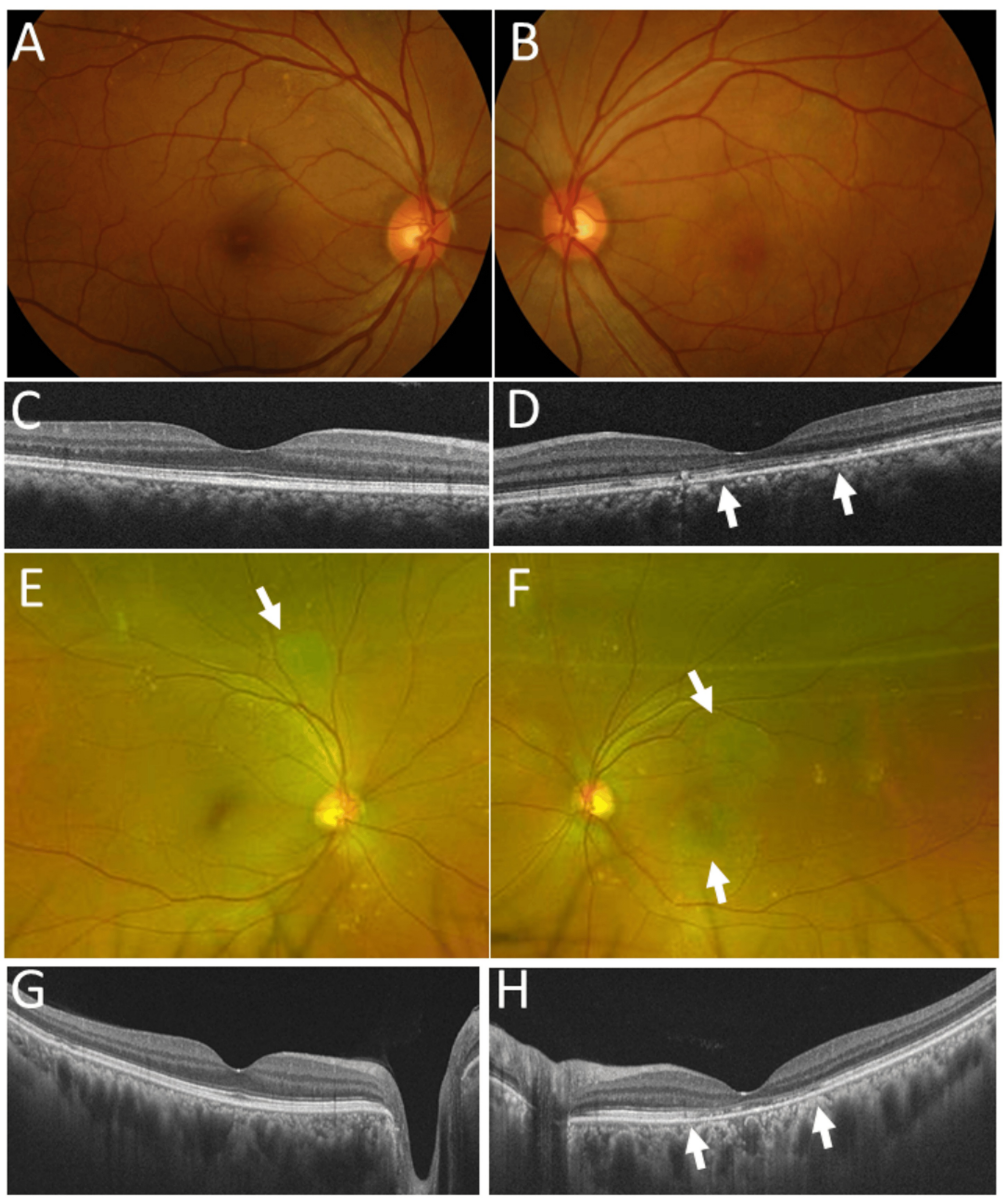
Fundus photographs and optical coherence tomography at age 45 and 60 years Fundus photographs (A, B) and horizontal section images of optical coherence tomography (C, D) in the right eye (A, C) and the left eye (B, D) at age 45 years. Wide-field fundus photographs (E, F) and horizontal section images of optical coherence tomography (G, H) in the right eye (E, G) and the left eye (F, H) at age 60 years. Note no serous retinal detachment and disrupted photoreceptor ellipsoid zones (between arrows) in the left eye (D, H), compared with normal photoreceptor ellipsoid zones in the right eye (C, G). Also note mild atrophic retinal areas as a sequel to serous retinal detachment (arrows, E, F)

Oral prednisolone and cyclosporine were slowly tapered over 10 years to 1 mg daily and discontinued at the age of 46 years. One month later, she experienced a relapse of nephrotic syndrome and restarted oral prednisolone 20 mg daily and cyclosporine 100 mg daily. At that time, there was no relapse of serous retinal detachment in either eye (Figures 5A-5D). Three years later, at age 49, oral cyclosporine, which had been tapered, was discontinued, while oral prednisolone, previously tapered to 1 mg daily, was increased again to 7 mg daily. At age 54, oral prednisolone was discontinued, and she remained free of proteinuria until the latest visit at age 61. The best-corrected visual acuity was 1.5 in both eyes, and she showed mild scar lesions in both eyes (Figures 5E-5H). She was healthy and asymptomatic, taking an oral combination of amlodipine 5 mg and atorvastatin 5 mg daily for hypertension and dyslipidemia (Table 1).

## Discussion

This report aims to explore the underlying causes of central serous chorioretinopathy-like manifestations in minimal change nephrotic syndrome. As summarized in the clinical course chart (Figure 2), the patient developed nephrotic syndrome at age 33 and experienced two major relapses at ages 36 and 41. She also had a minor relapse at age 37, five months after the major relapse at 36, and four additional minor relapses at ages 44, 46, 50, and 51. As summarized in Table 2, the onset of central serous chorioretinopathy-like manifestations, localized to the left eye, occurred three months after the initial onset of nephrotic syndrome at age 33. Two subsequent episodes of these manifestations in both eyes were observed five months and one month after the major relapses of nephrotic syndrome at ages 36 and 41, respectively.

**Table 2:** Summary of three episodes of central serous chorioretinopathy-like manifestations in relation to the timing of nephrotic syndrome and corresponding treatments

	33 years	36 years	41 years
Central serous chorioretinopathy	Onset	First relapse	Second relapse
Involved eyes	Left eye	Both eyes	Both eyes
Timing relative to onset and relapses of nephrotic syndrome	3 months later	5 months later	1 month later
Oral dose of prednisolone at the onset of serous retinal detachment	50 mg every other day	22.5 mg daily	30 mg daily
Oral dose of cyclosporine at the onset of serous retinal detachment	None	None	150 mg daily
Timing of remission of serous retinal detachment	3 months later	3 months later	6 months later
Oral dose of prednisolone at remission of serous retinal detachment	35 mg every other day	17.5 mg daily	5 mg daily
Oral dose of cyclosporine at remission of serous retinal detachment	None	None	40 mg daily

Regarding the dose of corticosteroids (Table 2), the onset of central serous chorioretinopathy-like manifestations at age 33 occurred while she was taking oral prednisolone 50 mg every other day, during the tapering of prednisolone from 50 mg daily following the first three-day course of methylprednisolone 500 mg daily. The relapse of central serous chorioretinopathy-like manifestations at age 36 occurred while she was taking oral prednisolone 22.5 mg daily, during the tapering of prednisolone from 40 mg daily after the second three-day course of methylprednisolone 500 mg daily. The second and final relapse of these manifestations at age 41 occurred while she was taking oral prednisolone 30 mg daily and cyclosporine 150 mg daily.

To date, three underlying clinical factors have been proposed to explain central serous chorioretinopathy-like manifestations in nephrotic syndrome. The first is that changes similar to those seen in minimal change disease in the glomeruli might occur in the choriocapillaris and retinal pigment epithelium, leading to leakage into the subretinal space [15,16]. The second is that hypoalbuminemia may induce osmotic changes, resulting in fluid accumulation in the subretinal space [17,18]. The third is that corticosteroids may damage the retinal pigment epithelium, causing leakage into the subretinal space [3].

Based on accumulated case reports, central serous chorioretinopathy-like manifestations have been observed at the onset of nephrotic syndrome in patients who had not received corticosteroids [19-23]. In other cases, patients with nephrotic syndrome developed these manifestations during corticosteroid therapy [24-28]. Our patient developed central serous chorioretinopathy-like manifestations three times while receiving corticosteroids: at the onset of nephrotic syndrome and during two major relapses. The timing in our patient suggests that these manifestations may be induced by corticosteroid administration during the course of nephrotic syndrome, as previously reported [3].

The thickened choroid, detectable by optical coherence tomography, is recognized as a clinical feature of central serous chorioretinopathy [29]. Recently, patients with nephrotic syndrome have also been shown to have increased central choroidal thickness on optical coherence tomography compared with healthy controls [16]. These findings suggest that central serous chorioretinopathy-like manifestations may arise from the same pathophysiology as nephrotic syndrome itself. The choriocapillaris of the eye may exhibit lesions similar to minimal change disease in the kidney [9]. This hypothesis of a shared mechanism between nephrotic syndrome and central serous choriocapillaris cannot be confirmed because biopsy of the choroid is not technically feasible. Consequently, no pathological examination has been performed in central serous chorioretinopathy [30]. From another perspective, corticosteroid administration has been identified as a risk factor for developing central serous chorioretinopathy in a population-based cohort study of nephrotic syndrome [15].

Nephrologists caring for patients with nephrotic syndrome monitor for visual symptoms, such as blurred vision, metamorphopsia, and photophobia, which may indicate the development of central serous chorioretinopathy-like manifestations. Conversely, ophthalmologists evaluating patients with central serous chorioretinopathy should consider nephrotic syndrome in the differential diagnosis. The kidney and eye can sometimes be affected simultaneously, not only by various inflammatory diseases [10-13], but also by degenerative diseases [31].

## Conclusions

This study describes a female patient who developed minimal change nephrotic syndrome at age 33 and was followed by a nephrologist and ophthalmologist at a single institution for 28 years, until age 61. The patient developed central serous chorioretinopathy-like manifestations in the left eye while receiving oral prednisolone, three months after the onset of nephrotic syndrome. She subsequently experienced these manifestations in both eyes twice, at ages 36 and 41, coinciding with major relapses of nephrotic syndrome. Afterward, she did not develop further central serous chorioretinopathy-like manifestations. She discontinued oral prednisolone at age 54 and remained free of nephrotic syndrome until the latest visit at age 61. The three episodes of central serous chorioretinopathy-like manifestations in this patient appear to be related to the onset and relapses of minimal change nephrotic syndrome itself. Corticosteroid therapy for nephrotic syndrome may also have contributed to the development of these ocular manifestations. The long-term, consistent temporal association between episodes of central serous chorioretinopathy and the onset and relapses of minimal change nephrotic syndrome is clearly supported by 28 years of longitudinal clinical observation. This parallel course suggests a possible shared pathophysiological mechanism or common triggering factors for both conditions.
